# HIV-2 diagnosis and quantification in high-risk patients

**DOI:** 10.1186/1742-6405-5-18

**Published:** 2008-08-14

**Authors:** Philip A Chan, Sarah E Wakeman, Timothy Flanigan, Susan Cu-Uvin, Erna Kojic, Rami Kantor

**Affiliations:** 1 Department of Internal Medicine, The Warren Alpert Medical School of Brown, University and Rhode Island Hospital, Providence, RI 02903, USA; 2 Division of Infectious Disease, The Warren Alpert Medical School of Brown University and The Miriam Hospital, Providence, RI 02906, USA

## Abstract

Current diagnostic assays for HIV-1 do not always test for the presence of HIV-2 in the United States. We present the case of a patient from Cape Verde, who was admitted to our hospital with rapidly deteriorating neurological function and multiple white matter lesions on MRI likely secondary to progressive multifocal leukoencephalopathy (PML). Initially, the patient had a positive EIA for HIV, but a negative HIV-1 Western Blot and no viral load detected on a branched-DNA assay. A repeat viral load by reverse transcriptase methodology (RT-DNA) detected 121,000 copies and an HIV-2 Western Blot was positive. The case highlights an extremely rare presentation of HIV-2 with severe neurological disease. We discuss the different tests available for the diagnosis and monitoring of HIV-2 in the United States.

## Background

HIV-2, the second AIDS-causing virus, is found predominantly in the Portuguese speaking countries of West Africa, with the highest rates of infection in Guinea-Bissau [1]. The prevalence of HIV-2 in the United States is extremely low [2], and the current guidelines recommend testing for HIV-2 only in the case of an indeterminate western blot or in patients with known links to West Africa [2,3]. While this screening practice may make sense for the majority of U.S. cities where the percentage of the population of West African descent is decidedly small, cities with a significant immigrant community from infected regions should consider increased surveillance. We present the case of a patient of Cape Verdean descent with likely PML in the setting of HIV-2, and discuss the difficulties of diagnosing HIV-2 in the United States.

## Case presentation

A 48 year-old male with a past medical history significant only for cataracts was admitted to our hospital with weakness, difficulty walking, and confusion that began one day prior to admission. In addition to the neurological symptoms, the patient had experienced a flu-like illness three to four weeks earlier accompanied by a ten to fifteen pound weight loss. The patient was afebrile with mental status changes and abnormal cerebellar findings on neurological exam including a wide-based gait, ataxia, dysmetria on finger-to-nose, and difficulty with rapid alternating hand movements. On further history, the patient took no medications and was born in Cape Verde, immigrating to the United States six years earlier. While he admitted to having multiple recent female sexual partners, he denied any drug use or any male sexual partners. His wife and five children remained in Cape Verde. His history raised the possibility of acute HIV infection or an AIDS-related illness.

An initial HIV enzyme immunoassay (EIA) was performed in the emergency room and returned positive (Bayer ADVIA Centaur HIV-1/O/2 EHIV EIA). Other laboratory tests were normal, aside from a slightly decreased white count of 3,800 cells/uL. A non-contrast CT of the brain on admission noted no acute abnormalities. An MRI with and without contrast of the brain was performed on the second day of admission, which showed multifocal supra and infratentorial T2 flare hyperintense lesions felt to be consistent with multiple sclerosis, an acute demyelinating process, or Lyme disease. Two lumbar punctures were subsequently performed showing four nucleated cells, an elevated protein of 103 mg/dL, and a normal glucose. Testing of the cerebrospinal fluid (CSF) was negative for cytomegalovirus (CMV, PCR), Epstein-Barr Virus (EBV, PCR), varicella zoster virus (VZV, PCR), Lyme (IgM and IgG antibodies, PCR), herpes simplex virus (HSV-1 and -2, IgM and IgG antibodies), Toxoplasmosis (IgM and IgG antibodies), India Ink, Cryptococcal antigen, Streptococcal antigen, rapid plasma reagin (RPR), JC Virus (PCR), acid-fast bacilli (AFB), and cytology. Other labs sent for the evaluation of mental status change were normal including electrolytes, B12, TSH, and a urine drug screen. Serum tests looking for an infectious etiology were also negative, including an RPR, fluorescent treponemal antibody (FTA-ABS), CMV antibodies, and Lyme antibodies. A hepatitis panel revealed past infections with hepatitis A and B, and a negative hepatitis C antibody. Blood, urine, and CSF cultures were negative, as was a rapid influenza. The patient's CD4 count returned at 202 (17%, ratio 0.3).

The patient improved with supportive care over the hospital course and was discharged on day five with follow-up to an outpatient clinic. Six days later the patient was seen at HIV clinic, at which point his confirmatory western blot for HIV-1 was still pending. Based on the patient's clinical history, CD4 count, positive EIA, and recent immigration from Western Africa, it was felt that the patient was most likely suffering from an HIV-related neurological process. He was started on the anti-retroviral regimen of ritonavir-boosted atazanavir (ATV), tenofovir (TDF), and emtricitabine (FTC), and a plasma viral load was sent.

Six days after the clinic visit, the patient was readmitted to the hospital for continued confusion and gait disturbance. The patient's initial confirmatory western blot for HIV-1 returned negative and the viral load was undetectable (branched DNA technology, Versant HIV-1 RNA 3.0, Bayer). Given the confusing picture and strong clinical suspicion for HIV, a second Western blot specific for HIV-2 was sent, as well as a repeat viral load using RT-PCR analysis (Roche Amplicor RT-PCR). The patient had a repeat MRI which showed interval worsening of the white matter lesions, but no new processes. During the course of this hospitalization, the patient's second viral load returned at 121,000 copies/mL and the western blot was positive for HIV-2. The differential diagnosis based on the patient's clinical history and imaging included PML, HIV encephalitis and/or lymphoma. Given the patient's negative CSF EBV and disseminated (non-solitary) MRI lesions, it was deemed highly unlikely that the patient had CNS Lymphoma. Although the patient had a negative JC virus PCR, review of the MRI found the lesions to be most consistent with PML and/or HIV encephalitis. A brain biopsy was considered, but the patient and his family refused.

Looking back over the hospital's records, it was discovered that the patient had been seen two years prior to this admission for a unilateral facial droop. In addition, a steadily declining WBC count was noted through several emergency room visits. Based on these findings, as well as the clinical presentation, the patient was felt to have chronic, as opposed to acute, HIV-2. While the patient may have had sexual contact with other West African immigrants in this country, it seemed most likely that he had become infected while living in Cape Verde more than six years earlier.

Over the first week of the second admission, the patient worsened neurologically, becoming incontinent and acutely agitated requiring medication with anti-psychotics. A repeat MRI of the brain on day eight of admission showed rapid progression of the white matter disease, as described above. Given the patient's poor prognosis, hospice was considered. The patient was changed to ritonavir-boosted lopinavir, and over the next couple weeks made significant clinical improvements, regaining continence, becoming increasingly lucid, and improving in gait and balance. By the fourth week of his admission, his CD4 count had increased to 331 (17.4%), although his MRI showed no regression of the lesions. The patient was discharged on HAART in stable condition, but with persistent neurological deficits.

## Discussion

We present a case report of an unusual and challenging diagnosis of HIV-2 in the United States. Our patient emigrated from Cape Verde, an archipelago off the west coast of Africa with an estimated population of 460,000. Cape Verde is a place defined by migration, with approximately 500,000 people living abroad, 265,000 of which are estimated to be in the United States [4]. The migration of Cape Verdeans to the United States began in the 1800's with whaling ships that carried West Africans to New England's shores. New Bedford, Massachusetts and Providence, Rhode Island are America's oldest Cape Verdean communities, with 1,592 legal immigrants settling in the Providence Metro area between 1991 and 1998 alone [5].

The prevalence of non-subtype B HIV in the United States is approximately 2% [6]. The virus isolated in this patient, HIV-2, is common in parts of Western Africa, but is rare in the United States [7]. HIV-2 is thought to have a milder disease course with a longer time to the development of AIDS than HIV-1 [8,9]. Clinical presentations of neurological syndromes with HIV-2 are extremely rare [10,11]. Since being discovered in 1985 [12], only 79 cases of HIV-2 have been reported in the United States with 52 of those patients having originated in Western Africa [2]. Given the low prevalence of HIV-2 in industrialized countries, the clinical course and optimal treatment strategies are unknown [12]. Non-nucleoside reverse transciptase inhibitors (NNRTI's) are not effective against HIV-2, whereas nucleoside reverse transciptase inhibitors (NRTI's) may be less effective [13,14]. Protease inhibitors have varying efficacy against HIV-2 [15-18] and use should be guided by genotype/phenotype profiles, not commercially available in the United States. Ritonavir-boosted atazanavir was initially started in this patient before the diagnosis of HIV-2, but was later changed to ritonavir-boosted lopinavir which has better efficacy against HIV-2 [16].

The identification of HIV-2 represents a diagnostic dilemma in the United States. The standard diagnosis of HIV-1 infection relies on a positive EIA followed by a confirmatory western blot assay in which two of the three HIV antigens (p24, gp41, and gp120) must be present. Screening EIA assays, including the newer rapid tests, are not always sensitive for detecting HIV-2 or group O HIV-1 [19-22], however the newer 4^th ^generation assays are better [5,23-25]. Routine western blots are specific mainly for HIV-1 antibodies and indeterminate western blots (i.e. detection of only one antigen, usually p24) may suggest infection with HIV-2. The only FDA-approved EIA assays that are able to detect HIV-2 are Abbott HIVAB HIV-1/2 (rDNA) EIA, Genetic Systems HIV-1/2 Peptide EIA, and Genetic Systems HIV-2 EIA.

Current CDC guidelines [26] state that HIV-2 serology should be checked in patients who: 1) Are from areas of high prevalence, mainly Western Africa; 2) Share needles or have sexual partners known to be infected with HIV-2 or are from endemic areas; 3) Received transfusions or other non-sterile medical care from endemic areas; 4) Are children of women with risk factors for HIV-2 infection. As sometimes clinical history is not available in patients with a high-suspicion for HIV infection and negative or indeterminate serology for HIV-1, additional testing should be performed for HIV-2.

Regarding viral RNA quantification, there are no FDA approved assays for the determination of HIV-2 viral load in the United States (Table 1). This creates a dilemma in the treatment of HIV-2 infected patients as viral loads are an integral part of patient monitoring. The five methods for detecting viral loads all routinely detect HIV-1 viral RNA from most group M subtypes, although small differences may exist in quantification capabilities [27-32]. Assays for HIV-2 are mainly developed for research purposes and none are commercially available [15,28,33-35]. The NucliSens EasyQ assay (BioMerieux, Netherlands) is approved for HIV-1 viral load quantification and has been shown to detect subtype A of HIV-2 by nucleic acid amplification [36]. Similarly, the Roche Amplicor assay was able to detect three of four HIV-2 samples [37]. Neither the branched DNA nor other RT-PCR assays have been shown to detect HIV-2, and none are approved by the FDA or regularly used to detect HIV-2. Differences between the assays are likely due to primers which are more likely to anneal and be specific to certain areas of both HIV-1 and HIV-2 depending on target sequences. Further studies are needed to define the sensitivity and specificity of these tests' ability to detect HIV-2.

**Table 1:** FDA approved assays for the quantification of HIV RNA

**Assay**	**Manufacturer**	**Technique**	**Sensitivity (copies/ml)**	**HIV-1 Subtypes**	**HIV-2**
AMPLICOR [39,40]	Roche	RT-PCR	50^†^-750,000	Group M (subtypes A-H)	Detected 3/4 HIV-2 [37]
Versant HIV-1 RNA 3.0 [41]	Bayer	bDNA	75–500,000	Group M (subtypes A-G)	No [42]
NucliSens HIV RNA QT [28,43]	BioMereiux	NASBA	176–3.4 million	Group M (not subtype G)	YES [36] (subtype A)
COBAS AmpliPrep, Taqman HIV-1 [44]	Roche	RT-PCR	48–10 million	Group M (subtypes A-H)	No [3,45]
RealTime HIV-1 [45]	Abbott	RT-PCR	40–10 million	Group M, N, O, recombinants	No [45]

NASBA: Nucleic acid sequence based amplification assay

RT-PCR: Reverse transcription polymerase-chain reaction

bDNA: Branched DNA assay

^†^The standard assay can detect 400–750,000 copies/ml and the ultra-sensitive assay can detect 50–100,000 copies/ml.

In our institution, patients are tested for HIV using the Bayer ADVIA Centaur HIV-1/O/2 EHIV EIA [38]. This was positive in our patient, but a western blot for HIV-1 was negative. The Versant branched DNA assay is our standard measure for HIV viral loads, but this technique did not quantify any viral RNA in this particular patient. Follow-up testing with a western blot specific for HIV-2 was positive and subsequent viral quantification based the Roche Amplicor system showed a significant viral load.

Our patient exemplifies the diagnostic difficulties of identifying HIV-2 in the United States. Fortunately, we were able to elucidate a history of Western African origin from our patient. All physicians involved in screening for HIV should be aware of the limitations between assays and know which test their institution uses. Clinicians need to have a high index of suspicion in patients with risk factors for HIV-2 to appropriately diagnose and treat the disease.

## Competing interests

SC reports receiving grant support for an unrelated study from Bristol-Myers Squibb. All other authors declare there are no competing interests in this work. The present study was unfunded.

## Authors' contributions

PC and SW participated in the research, writing, and editing of the manuscript. TF, SC, EK, and RK participated in the writing and editing of the manuscript. All authors read and approved the final manuscript.
